# Feline Blood Groups: A Systematic Review of Phylogenetic and Geographical Origin

**DOI:** 10.3390/ani11123339

**Published:** 2021-11-23

**Authors:** Alessandra Gavazza, Giacomo Rossi, Maria Teresa Antognoni, Matteo Cerquetella, Arianna Miglio, Sara Mangiaterra

**Affiliations:** 1 School of Biosciences and Veterinary Medicine, University of Camerino, 62032 Camerino, Italy; alessandra.gavazza@unicam.it (A.G.); giacomo.rossi@unicam.it (G.R.); matteo.cerquetella@unicam.it (M.C.); 2 Blood Bank and Transfusion Unit EMOVET-UNIPG, Department of Veterinary Medicine, University of Perugia, 06126 Perugia, Italy; maria.antognoni@unipg.it (M.T.A.); miglioarianna@libero.it (A.M.)

**Keywords:** cat breed, blood groups, phylogenetic, geographical origin

## Abstract

**Simple Summary:**

Numerous breeds have been identified in the domestic cat, grouped according to their phylogenetic or geographical origin. In the cat, the AB blood group system is the most important feline system, and the determination of the blood group by specific methods is an essential step to avoid or reduce the risk of an adverse reaction in the recipient patient. Many studies have been published on the distribution and prevalence of blood types in pedigree and non-pedigree cats, but the information has never been collated in a systematic manner.

**Abstract:**

Domestic cats descended from the African wildcat several thousand years ago. Cats have spread to all parts of the world, probably along routes between civilizations or geographical boundaries, leading to the movement of species, from Asia to the African continent through the Mediterranean basin, and finally to the American continent, Australia, and New Zealand. Currently, 73 cat breeds are recognized by the International Cat Association. With the increasing interest in the selection of breeds, the determination of blood groups in cats has acquired importance over time. The AB blood group system is the most important blood system in cats, in which A, B, and AB or C blood groups are identified. This systematic review describes data from previously published reports about cat blood types and cat breeds. After applying specific criteria, 28 eligible studies were identified in which the prevalence percentages for each blood group in correlation with specific cat breeds were reported. The breeds were, in turn, divided into four groups according to their geographic and phylogenetic origins as follows: Asian cat breeds, American cat breeds, European cat breeds, and breeds from Oceania. Although numerous studies were carried out before 2021, gaps in the literature on the AB system and, in particular, the Mik group are highlighted.

## 1. Introduction

Over the centuries, numerous breeds have been identified in the cat “species”, grouped according to the International Cat Association, which recognizes 73 breeds [1]. Cats breeds can be grouped according to their phylogenetic and geographical origins. Studies showed that cat breeds developed from the Mediterranean, Western European, Arabian, and Asian areas based on selecting certain phenotypes from adapted populations [2]. For this reason, many cat breeds are still genetically close to the landrace cats they were developed from. In 2008, a study determined genetic differentiation by analysis of molecular variance for cat breeds originating from Europe, the Americas, East Africa, the Mediterranean, and Asia [2]. Genetic differences have been analyzed in the worldwide cat population showing differences in mitochondrial DNA [3] and single nucleotide polymorphisms [4]. In accordance with these results, the prevalence of blood groups varies among feline breeds and consequently among phylogenetic origin [5]. In humans, the susceptibility to diseases linked with ABO blood groups has been shown, such as cancer, cardiovascular diseases, and infections [6]. Studies showed that blood type O predisposes patients to gastrointestinal infections such as *Escherichia coli* and *Vibrio cholerae* [7,8]. This blood type showed higher susceptibility to peptic ulcers correlated with *Helicobacter pylori* infection [7,9], and also *Helicobacter pylori* attachment to the human gastric mucosa was mediated by specific fucosylated antigens [7,9]. Blood type A was associated with a high incidence of *Pseudomonas aeruginosa* infection; blood type B and AB are associated with increased incidence of tuberculosis, gonorrhea, *Streptococcus pneumoniae*, *Salmonella*, and *E. coli* infections [6]. In the cat, the AB blood group system is the most important feline system [10], where the presence of specific gangliosides characterize the different blood types [11,12]. As in humans and other mammals, studies showed the correlation between the blood type in cats and the neuraminic acid residues present on the surface of erythrocytes [13]. The Cytidine monophospho-N-acetylneuraminic acid hydroxylase (*CMAH*) was the first gene shown to control blood types in non-primate mammals correlating with the production of the sialic acids on red blood cells [14,15,16]. The feline *CMAH* is the gene shown to control blood types in cats; this gene codes for the *CMAH* enzyme and determines the type of sialic acid on erythrocytes [14,16]. The *CMAH* enzyme is active in type A cats, which causes N-acetylneuraminic acid and N-glycolylneuraminic conversion of N-acetylneuraminic acid (NeuAc) in N-glycolylneuraminic (NeuGc), while it is nonfunctional in type B cats [14,16]. The NeuGc is mainly present in the type A group with a minor amount of NeuAc and two intermediate forms, while the type B cat has only NeuAc [14]. Cats with blood type AB (or C) present both NeuGc and NeuAc sialic acids at low levels on the red blood cell surface that could result from a reduction of *CMAH* activity [14,17]. Blood types are inherited according to Mendelian law, with A being dominant over B. According to the combination of dominant and recessive alleles, feline blood groups are classified into A, B, and AB groups [10]. The A blood group is associated with *A*/*A*, *A*/*b,* and *A*/*a^c^* genotypes; the B blood group is correlated with *b*/*b* genotype; and the AB blood group with *a^c^*/*a^c^* or *a^c^*/*b* genotypes [18,19,20]. In 2007, a distinct alloantibody named Mik was identified using standard tube and novel gel column cross-matching methods [21]. However, the incidence of the Mik antigen in the feline population is not known.

Recently, in a study conducted in 2021, cats were evaluated for the presence of naturally occurring anti-A and anti-B alloantibodies (NOAb), suggesting the presence of new feline erythrocyte antigens (FEA) [22].

Cats that receive an incompatible blood type may develop adverse reactions such as hemolysis, vomiting, or pyrexia and neonatal isoerythrolysis [5,23]; for this reason, blood group determination is an important practice in veterinary medicine.

As in dogs, to determine the DEA group [24], several methods have been proposed to determine the blood group in cats [5,25]. Feline blood typing can be performed: (i) by a point-of-care test (CARD) consisting of a card with wells that contain lyophilized monoclonal anti-A or anti-B antibody [26]; (ii) by a point-of-care test (CHROM) based on immunochromatographic diffusion of RBCs with monoclonal anti-A and anti-B antibodies (DME VET A + B, provided by Alvedia, Lyon, France) [27,28]; (iii) by a technique (GEL) method based on gel columns containing anti-A antibodies [29]; (iv) by a SLIDE test and tube assay (TUBE) in which the degree of agglutination is scored as for the CARD method [30]. Many studies have been published on the distribution and prevalence of blood types in pedigree and non-pedigree cats. However, the information has never been collated in a systematic manner. Systematic reviews differ from traditional reviews; this is due to a systematic search method of the literature, which implies a detailed and comprehensive plan and search strategy by identifying, appraising, and synthesizing all relevant studies on a particular topic [31,32].

A systematic attempt [31,32] has been made to identify all the published data and to summarize the information to define the distribution of blood groups in different areas of the world, and to evaluate the prevalence according to phylogeographical origin.

## 2. Materials and Methods

### 2.1. Systematic Search Strategy

A comprehensive literature search was conducted systematically using three English databases, PubMed (National Library of Medicine, 8600 Rockville Pike, Bethesda, MD 20894, USA), ScienceDirect (Elsevier B.V., Amsterdam, The Netherlands), and Google Scholar (Google Inc., Mountain View, CA, USA), for articles published prior to 2021. We also manually searched the reference lists of the selected studies and relevant reviews. The searching process was accomplished using a combination of descriptors, synonyms, and combinations of several search terms, including “Cat blood groups”, “Feline blood groups”, “Cat blood types”, “Feline blood types”, “Cat blood methods”, “Feline blood methods”, “Cat blood typing”, “Feline blood typing”, “Cat blood prevalence”, and “Cat breed blood prevalence” (Table 1). All citations were downloaded into EndNote. For all selected studies, the following data were extracted: year of publication, country of study, methods for blood typing, breeds of cats, and phylogeographical origins of cats.

### 2.2. Study Selection

The inclusion criteria included articles that provided descriptions of blood groups in cats and the prevalence of feline blood groups in a given country or in a breed. The search was restricted to the English language; manuscripts written in other languages were excluded. The exclusion criteria also included studies with insufficient data or that involved other animal species or humans. Finally, scientific articles for which it was not possible to obtain the complete text or to access data about the prevalence in the breeds included have been excluded (Table 2).

This literature review followed the PRISMA (Preferred Reporting Items for Systematic reviews and Meta-Analyses) flowchart and is based on PRISMA’s statement [31].

### 2.3. Data and Quality Assessment

From each study, data concerning the year of publication, the country where the cats were enrolled, the prevalence of specific feline blood groups, the blood-typing method used, and the breed of cats were recorded. The selected studies were divided into 4 groups—A, B, AB, and Mik (+/−)—representing the blood groups. The selected studies were also divided into 4 groups based on the phylogeographical origin of each breed, Asia, the Americas, Europe, and Oceania.

## 3. Results

### 3.1. Study Selection

**Figure 1:**
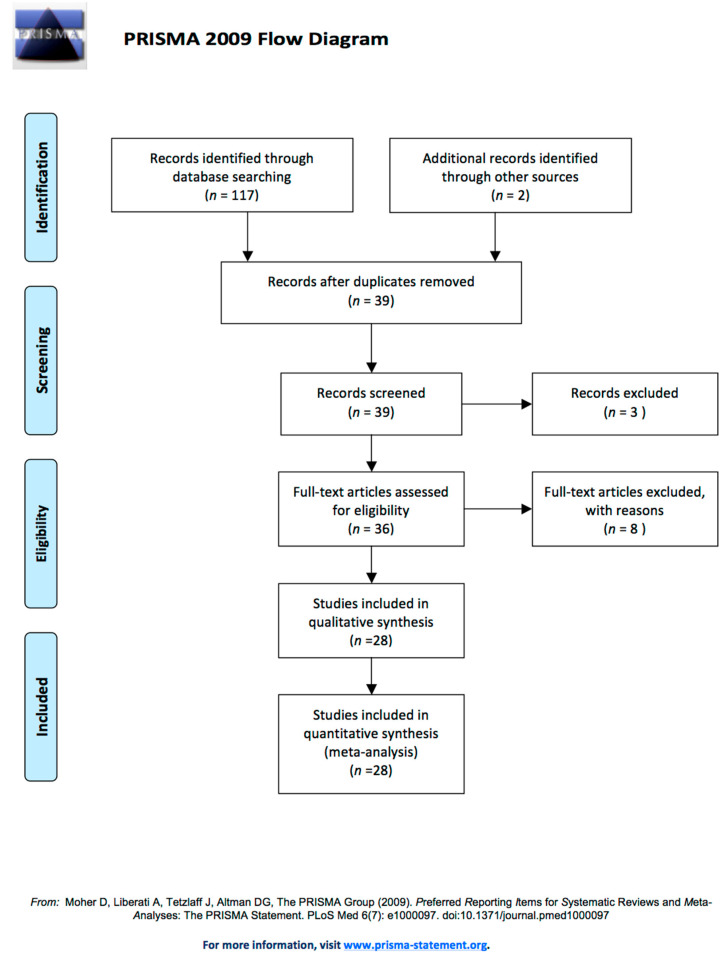
This literature review followed the PRISMA (Preferred Reporting Items for Systematic reviews and Meta-Analyses) flowchart. www.prisma-statement.org (accessed on 20 November 2021).

After the research strategy was applied, 119 records were shortlisted from the initial search. Full texts of the identified articles were assessed to produce the final selection of articles included for this systematic review. In total, 80 articles were excluded as duplicates or because it was not possible to obtain the complete text, and from the remaining 39 studies, 11 were finally excluded because they were written in a non-English language and were without data about the prevalence in breeds. In conclusion, 28 articles were included in this review (Figure 1).

### 3.2. Feline Blood Groups’ Prevalence Based on Natural Phylogeographical Origin

After the application of the criteria and duplicates were removed, 28 eligible studies were identified. According to the natural phylogeographical origin, breeds were divided into four groups: Asian cat breeds, American cat breeds, European cat breeds, and Oceanian cat breeds. From the number of cats studied for each breed, the prevalence percentage of blood groups divided into A, B, AB, and Mik was calculated or transcribed (Table 3).

According to the genetic data: nine different breeds were collected for Asian breeds (Abyssinian, Bengal, Birman, Bumbay, Burmese, Exotic shorthair, Persian, Siamese, and Somali); two breeds were reported among American cat breeds (Maine Coon and Ragdoll); 10 breeds were reported for European cat breeds (Turkish Van, Chartreux, British Short Hair, Siberian Forest, Scottish Fold, Russian Blue, Norwegian Forest, Devon Rex, Sphinx, and European Short Hair/long hair). For each phylogenetic group, non-pedigree cats were also reported.

A total of 2415 cats were included in the Asian breeds; among these cats, A and B blood groups were identified in all studies, the AB group was identified in 32 studies, and none of the studies reported the Mik research.

For the American cat breeds, 1998 animals were studied; among these, all studies reported A and B blood group identification, and eight studies reported the AB blood type identification, and none reported the Mik identification.

A total of 4914 cats were studied in breeds of European origin; among these A and B blood groups were identified in all studies, the AB group was identified in 38 studies, and only one study reported the Mik type.

Finally, among cat breeds from Oceania, 432 animals were studied for blood groups. A blood group was identified in all studies; B and AB blood types were reported in two studies, and none reported Mik identification.

**Figure 2:**
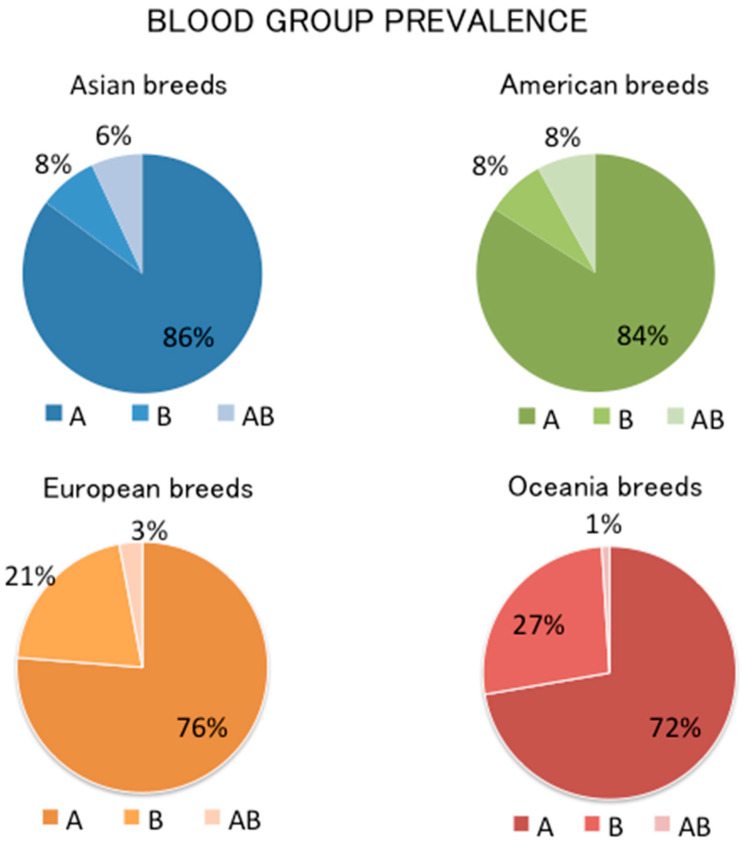
Prevalence of cat blood groups.

The means of the prevalence rates for each blood group have been summarized in the graphs below (Figure 2)

## 4. Discussion

Domestic cats have their origins in Old World wildcats. The earliest evidence of the cat-human relation was found in Cyprus and determined to be about 9000 years old; subsequently, numerous historical finds belonging to the populations of ancient Egypt have shown the sacred role of this animal [61,62]. Over the centuries, cat genotypes and phenotypes have been influenced by geographical and human history, from the Neolithic Period when agriculture emerged in human societies to the modern era with the commercial relationships between continents [60]. For example, common characters in cats in the USA and Canada are the same in cats from the British isles, due perhaps, to their trans-Atlantic migrations from the fifteenth century onward. Cats present in Australia, the south and center of Africa, and South America show genetic aspects similar to the European domestic cat, probably due to the intense displacements of colonizers and trade routes starting from the fifteenth century [61]. Furthermore, the presence of geographical barriers, such as high mountain ranges (for example, the Pyrenees or the Alps), and islands (such as Iberia or San Marcos island in California), have represented a pool of selection of specific genotypic characters that have characterized the development of some cat breeds [63]. Today, cats are present on all continents except in the most remote regions of the world, for example, Antarctica. Several studies have shown the distribution and prevalence of blood types in pedigree and non-pedigree cats; however, there is no evidence of the relationship between blood groups and phylogenetic origin. In humans, a different distribution of blood types around the world was suggested [63]. For example, it was shown that the A blood type was most common in Europeans, the B blood group in Asian populations, and O in South Americans. The frequency of the Rh-negative phenotype differs between populations: in Africa and Asia, the Rh-negative phenotype is less common; meanwhile, Western nations, such as Britain and the USA, have Rh factor negativity lower than the Saudi Arabian population [63]. The surface antigens that determine blood group influence the natural resistance of people to many infectious diseases [64]. Studies showed that people with blood group AB are most sensitive to infectious diseases because they carry all antigens on their cells [64]. In veterinary medicine, studies have reported that feline infectious peritonitis (FIP) is more common in purebred cats such as the Abyssinian, Bengal, Birman, Himalayan, Ragdoll, and Rex [65]. Today, the correlation between blood group and predisposition to infectious diseases caused by bacteria or viruses in cats (such as FIV, FeLV, or FIP) has not yet been fully demonstrated.

In this systematic review, 27 articles on the prevalence of cat blood groups were included. Selected scientific manuscripts included both purebred and non-pedigreed cats. They were divided into four groups based on the origin of the breeds in cats of Asian, American, European, and Oceanian origin (including Australia and New Zealand). For each scientific study, we obtained the prevalence of the blood group divided into A, B, AB, and Mik. The means of the prevalence rates for each blood group showed that group A was high in all cats, 86% in Asian breeds, 84% in American breeds, 76%in European breeds, and 72% in breeds from Oceania), while group B prevalence was higher in European and Oceanian breeds (respectively, 21% and 27%) compared with Asian and American breeds (8% and 8%, respectively). The means of the prevalence rate of group AB was lower in all cats (6% in Asian breeds, 8% in American breeds, 3% in European breeds, and 1% in the breed from Oceania). The similarities in Asian and American breeds could be explained by the geographical proximity and trade relations between the two continents. Blood group prevalence of breeds from the European continent and Oceania could be due to the developed relationships Europe had with Australia and New Zealand, starting from the first English colonies in the 17th century.

## 5. Conclusions

The role of AB blood groups in cats is important in veterinary transfusion medicine. As with dogs, the determination of the blood group by specific methods is an essential step to avoid or reduce the risk of an adverse reaction in the recipient patient. According to our results, blood group types A, B, and AB in cats have been determined in all continents, while the role of the Mik antigen remains unclear. Recently, NOAb outside the AB system has been proposed in cats suggesting the presence of new possible antigens. Furthermore, in cats, the predisposition of some breeds to infectious diseases is known. Future studies should evaluate the possible correlation between blood group and the prevalence of infectious diseases, as has been demonstrated in humans.

## Figures and Tables

**Table 1:** Literature search databases and descriptors used for the systematic search strategy.

Databases	Descriptors
Pubmed ScienceDirect Google Scholar	“Cat blood groups”
	“Feline blood groups”
	“Cat blood types”
	“Feline blood types”
	“Cat blood methods”
	“Feline blood methods”
	“Cat blood typing”
	“Feline blood typing”
	“Cat blood prevalence”
	“Cat breed blood prevalence”

**Table 2:** Literature search criteria.

Criteria	
Inclusion	English language Cat blood groups Feline blood type prevalence
Exclusion	Non-English language Other species blood groups Other animals blood types prevalence Full text not found

**Table 3:** Prevalence of cat blood groups (A, B, AB, and Mik) for each breed divided by phylogenetic origin in Asian (**a**), American (**b**), European (**c**), and Oceanian (**d**) cat breeds.

**(a) Blood group prevalence in Asian cat breeds.**						
**Breed**	***n***	**A%**	**B%**	**AB %**	**Mik**	**Reference**
Abyssinian	230	86	14	0	-	Giger et al., 1991 [33]
Abyssinian	2	50	0	50	-	Knottenbelt et al., 1999 [34]
Abyssinian	6	100	0		-	Bagdi et al., 2001 [35]
Abyssinian	20	89	11	0	-	Malik et al., 2005 [36]
Abyssinian	194	80	20		-	Giger et al., 1991 [33]
Abyssinian	6	66	33	0	-	Spada et al., 2014 [37]
Abyssinian	20	100	0		-	Jensen et al., 1994 [38]
Bengal	8	50	0	50	-	Knottenbelt et al., 1999 [34]
Bengal	7	86	14	0	-	Forcada et al., 2007 [39]
Birman	2	100	0	0	-	Fosset et al., 2014 [40]
Birman	24	62	30	8	-	Knottenbelt et al., 1999 [34]
Birman	4	50	50	0	-	Malik et al., 2005 [36]
Birman	7	100	0	0	-	Spada et al., 2014 [37]
Birman	216	82	18		-	Giger et al., 1991 [33]
Birman	5	40	60		-	Jensen et al., 1994 [38]
Bombay	1	100	0	0	-	Knottenbelt et al., 1999 [34]
Burmese	10	90	10	0	-	Knottenbelt et al., 1999 [34]
Burmese	9	100	0		-	Jensen et al., 1994 [38]
Burmese	30	93	3	3	-	Malik et al., 2005 [36]
Burmese	10	90	10	0	-	Knottenbelt et al., 1999 [34]
Burmese	5	100	0	0	-	Forcada et al., 2007 [39]
Exotic shorthair	3	100	0	0	-	Spada et al., 2014 [37]
Exotic shorthair	1	100	0		-	Jensen et al., 1994 [38]
Persian	8	100	0	0	-	Fosset et al., 2014 [40]
Persian	230	90	9.6		-	Giger et al., 1991 [33]
Persian	56	94	6		-	Jensen et al., 1994 [38]
Persian	5	100	0	0	-	Karadjole et al., 2016 [41]
Persian	17	88	11	0	-	Knottenbelt et al., 1999 [34]
Persian	8	50	12	37	-	Spada et al., 2014 [37]
Persian	170	75	25		-	Giger et al., 1991 [33]
Persian	9	66	33		-	Bagdi et al., 2001 [33]
Persian	7	85	0	14	-	Silvestre-Ferreira et al., 2004 [42]
Persian	9	67	22	11	-	Malik et al., 2005 [36]
Persian	5	80	20	0	-	Forcada et al., 2007 [39]
Siamese	4	100	0	0	-	Fosset et al., 2014 [40]
Siamese	4	100	0	0	-	Knottenbelt et al., 1999 [34]
Siamese	3	100	0	0	-	Spada et al., 2014 [37]
Siamese	3	100	0		-	Jensen et al., 1994 [38]
Siamese	3	100	0		-	Bagdi et al., 2001 [33]
Siamese	12	100	0	0	-	Malik et al., 2005 [36]
Siamese	19	100	0	0	-	Silvestre-Ferreira et al., 2004 [42]
Siamese	13	100	0	0	-	Forcada et al., 2007 [39]
Somali	9	77	0	22	-	Knottenbelt et al., 1999 [34]
Somali	27	77	23		-	Giger et al., 1991 [33]
Somali	9	100	0		-	Jensen et al., 1994 [38]
No pedigree	482	96	17	0	-	Ban et al., 2008 [43]
No pedigree	213	69	14	16	-	Merbl et al., 2011 [44]
No pedigree	8	100	0		-	Bagdi et al., 2001 [35]
No pedigree	262	88	11	0.4	-	Zheng et al., 2011 [45]
Total cats (n)	2415					
**(b) Blood group prevalence in American cat breeds**						
**Breed**	***n***	**A%**	**B%**	**AB %**	**Mik**	**Reference**
Maine Coon	75	100	0	0	-	Spada et al., 2014 [37]
Maine Coon	3	100	0		-	Jensen et al., 1994 [38]
Ragdoll	25	68	8	24	-	Spada et al., 2014 [37]
Ragdoll	5	80	20	0	-	Malik et al., 2005 [36]
Ragdoll	7	71	28	0	-	Knottenbelt et al., 1999 [34]
Ragdoll	61	77	4.9	18	-	Proverbio et al., 2013 [46]
No pedigree	178	94	9	0.6	-	Fosset et al., 2014 [40]
No pedigree	1072	99	1		-	Giger et al., 1991 [33]
No pedigree	400	96	4	0	-	McDermott et al., 2020 [47]
No pedigree	172	94	2.9	2.3	-	Medeiros et al., 2008 [48]
Total cats (n)	1998					
**(c) Blood group prevalence in European cat breeds**						
**Breed**	***n***	**A%**	**B%**	**AB %**	**Mik**	**Reference**
Turkish Van	78	42	57	0	-	Arikan et al., 2004 [49]
Turkish Van	1	100			-	Knottenbelt et al., 1999 [34]
Chartreux	5	100	0	0	-	Spada et al., 2014 [37]
British Short Hair	6	100	0	0	-	Spada et al., 2014 [37]
British Short Hair	8	100	0	0	-	Fosset et al., 2014 [40]
British Short Hair	30	66	33		-	Jensen et al., 1994 [38]
British Short Hair	10	90		10	-	Karadjole et al., 2016 [41]
British Short Hair	1	100	0		-	Bagdi et al., 2001 [35]
British Short Hair	85	41	59		-	Giger et al., 1991 [33]
British Short Hair	121	39	58	1.6	-	Knottenbelt et al., 1999 [34]
British Short Hair	5	40	60	0	-	Forcada et al., 2007 [39]
No pedigree	301	73	24	2	-	Arikan et al., 2006 [50]
No pedigree	240	72	25	2	-	Arikan et al., 2010 [51]
No pedigree	231	89	10	0.4	-	Barrot et al., 2017 [52]
No pedigree	312	72	25	2	-	Gurkan et al., 2005 [53]
No pedigree	105	98	1.9		-	Jensen et al., 1994 [38]
No pedigree	137	84	14	0.7	-	Juvet et al., 2011 [54]
No pedigree	30	96		3	-	Karadjole et al., 2016 [41]
No pedigree	97	88	7	4	-	Silvestre-Ferreira et al., 2004 [42]
No pedigree	196	10	91	0	-	Spada et al., 2020 [55]
No pedigree	131	12	95	0	-	Spada et al., 2020 [55]
No pedigree	73	100	0		-	Bagdi et al., 2001 [35]
No pedigree	112	77	17	6	-	Tasker et al., 2014 [20]
No pedigree	145	86	7	3.1	-	Di Tommaso et al., 2020 [56]
Siberian Forest	3	100	0	0	-	Spada et al., 2014 [37]
Scottish Fold	27	85	15		-	Giger et al., 1991 [33]
Scottish Fold	7	100	0	0	-	Fosset et al., 2014 [40]
Russian Blue	5	80	20	0	-	Malik et al., 2005 [36]
Russian Blue	1	100	0	0	-	Spada et al., 2014 [37]
Norwegian Forest	7	100	0	0	0	Spada et al., 2014 [37]
Norwegian Forest	2	100	0		-	Jensen et al., 1994 [38]
Devon Rex	6	50	50	0	-	Spada et al., 2014 [37]
Devon Rex	288	50	49	0	-	Giger et al., 1991 [33]
Devon Rex	100	57	43		-	Giger et al., 1991 [33]
Devon Rex	71	45	54	1	-	Malik et al., 2005 [33]
Devon Rex	2	100	0	0	-	Knottenbelt et al., 1999 [34]
Sphinx	7	71	28	0	-	Spada et al., 2014 [37]
European Short Hair/long hair	195	92	5	2.6	-	Spada et al., 2014 [37]
European Short Hair	125	88	8	4	-	Knottenbelt et al., 1999 [34]
European Short Hair/long hair	515	94	2	0.4	-	Marques et al., 2011 [57]
European Long Hair	14	78	7	14	-	Knottenbelt et al., 1999 [34]
European Short Hair/long hair	207	78	20	1.4	-	Mylonakis et al., 2001 [58]
European Short Hair/long hair	320	83	14	1.9	-	Nectoux et al., 2019 [59]
European Short Hair	132	90	3.8	6	-	Silvestre-Ferreira et al., 2004 [42]
European Long Hair	15	80	6.7	13	-	Silvestre-Ferreira et al., 2004 [42]
European Short Hair	95	65	33	2	-	Forcada et al., 2007 [39]
European Long Hair	10	90	10	0	-	Forcada et al., 2007 [39]
Total cats (n)	4614					
**(d) Blood group prevalence in Oceanian cat breeds**						
**Breed**	***n***	**A%**	**B%**	**AB %**	**Mik**	**Reference**
New Zealand No pedigree	89	79	20	1	-	Cattin, 2016 [60]
New Zealand No pedigree	156	89			-	Cattin, 2016 [60]
Australia No Pedigree	187	62	36	1.6	-	Malik et al., 2005 [35]
Total cats (n)	432					

## Data Availability

Not applicable.
